# Micelle-directed self-assembly of single-crystal-like mesoporous stoichiometric oxides for high-performance lithium storage

**DOI:** 10.1093/nsr/nwae054

**Published:** 2024-02-06

**Authors:** Yanhua Wan, Changyao Wang, Xingmiao Zhang, Yang Yin, Mengmeng Liu, Bing Ma, Linlin Duan, Yuzhu Ma, Wei Zhang, Changlin Zheng, Dongliang Chao, Fei Wang, Yongyao Xia, Wei Li

**Affiliations:** Department of Chemistry, Laboratory of Advanced Materials, Shanghai Key Laboratory of Molecular Catalysis and Innovative Materials, State Key Laboratory of Molecular Engineering of Polymers, College of Chemistry and Materials Science, Fudan University, Shanghai 200433, China; Department of Chemistry, Laboratory of Advanced Materials, Shanghai Key Laboratory of Molecular Catalysis and Innovative Materials, State Key Laboratory of Molecular Engineering of Polymers, College of Chemistry and Materials Science, Fudan University, Shanghai 200433, China; Department of Chemistry, Laboratory of Advanced Materials, Shanghai Key Laboratory of Molecular Catalysis and Innovative Materials, State Key Laboratory of Molecular Engineering of Polymers, College of Chemistry and Materials Science, Fudan University, Shanghai 200433, China; State Key Laboratory of Surface Physics and Department of Physics, Fudan University, Shanghai 200433, China; Department of Chemistry, Laboratory of Advanced Materials, Shanghai Key Laboratory of Molecular Catalysis and Innovative Materials, State Key Laboratory of Molecular Engineering of Polymers, College of Chemistry and Materials Science, Fudan University, Shanghai 200433, China; Department of Chemistry, Laboratory of Advanced Materials, Shanghai Key Laboratory of Molecular Catalysis and Innovative Materials, State Key Laboratory of Molecular Engineering of Polymers, College of Chemistry and Materials Science, Fudan University, Shanghai 200433, China; Department of Chemistry, Laboratory of Advanced Materials, Shanghai Key Laboratory of Molecular Catalysis and Innovative Materials, State Key Laboratory of Molecular Engineering of Polymers, College of Chemistry and Materials Science, Fudan University, Shanghai 200433, China; Department of Chemistry, Laboratory of Advanced Materials, Shanghai Key Laboratory of Molecular Catalysis and Innovative Materials, State Key Laboratory of Molecular Engineering of Polymers, College of Chemistry and Materials Science, Fudan University, Shanghai 200433, China; Department of Chemistry, Laboratory of Advanced Materials, Shanghai Key Laboratory of Molecular Catalysis and Innovative Materials, State Key Laboratory of Molecular Engineering of Polymers, College of Chemistry and Materials Science, Fudan University, Shanghai 200433, China; State Key Laboratory of Surface Physics and Department of Physics, Fudan University, Shanghai 200433, China; Department of Chemistry, Laboratory of Advanced Materials, Shanghai Key Laboratory of Molecular Catalysis and Innovative Materials, State Key Laboratory of Molecular Engineering of Polymers, College of Chemistry and Materials Science, Fudan University, Shanghai 200433, China; Department of Chemistry, Laboratory of Advanced Materials, Shanghai Key Laboratory of Molecular Catalysis and Innovative Materials, State Key Laboratory of Molecular Engineering of Polymers, College of Chemistry and Materials Science, Fudan University, Shanghai 200433, China; Department of Chemistry, Laboratory of Advanced Materials, Shanghai Key Laboratory of Molecular Catalysis and Innovative Materials, State Key Laboratory of Molecular Engineering of Polymers, College of Chemistry and Materials Science, Fudan University, Shanghai 200433, China; Department of Chemistry, Laboratory of Advanced Materials, Shanghai Key Laboratory of Molecular Catalysis and Innovative Materials, State Key Laboratory of Molecular Engineering of Polymers, College of Chemistry and Materials Science, Fudan University, Shanghai 200433, China

**Keywords:** mesoporous materials, micelles, single-crystal-like, Li_2_TiSiO_5_, lithium-ion batteries

## Abstract

Due to their uncontrollable assembly and crystallization process, the synthesis of mesoporous metal oxide single crystals remains a formidable challenge. Herein, we report the synthesis of single-crystal-like mesoporous Li_2_TiSiO_5_ by using soft micelles as templates. The key lies in the atomic-scale self-assembly and step-crystallization processes, which ensure the formation of single-crystal-like mesoporous Li_2_TiSiO_5_ microparticles via an oriented attachment growth mechanism under the confinement of an *in-situ* formed carbon matrix. The mesoporous Li_2_TiSiO_5_ anode achieves a superior rate capability (148 mAh g^−1^ at 5.0 A g^−1^) and outstanding long-term cycling stability (138 mAh g^−1^ after 3000 cycles at 2.0 A g^−1^) for lithium storage as a result of the ultrafast Li^+^ diffusion caused by penetrating mesochannels and nanosized crystal frameworks (5–10 nm). In comparison, bulk Li_2_TiSiO_5_ exhibits poor rate capability and cycle performance due to micron-scale diffusion lengths. This method is very simple and reproducible, heralding a new way of designing and synthesizing mesoporous single crystals with controllable frameworks and chemical functionalities.

## Teaser

Single-crystal-like mesoporous Li2TiSiO5 is synthesized via soft micelle directed self-assembly strategy, which enables the ultrafast Li+ diffusion and storage for safe lithium ion batteries.

## INTRODUCTION

Benefiting from open porous frameworks, high surface areas and large porosities, mesoporous metal oxide materials have attracted great scientific interest in the past two decades, especially in the field of sensors, catalysis, energy storage and conversion [1–6]. It has been well demonstrated that the performance of mesoporous metal oxides is not only affected by the porous structures but also highly depends on the crystal phase and crystalline degree of the frameworks [7–12]. In lithium-ion storage, microstructured single-crystal electrode materials show great advantages for ionic conductivity because they remove grain boundaries from inside materials, but usually trade off the diffusion distance of Li ions in the microsized particle, consequently reducing the rate capability and cycle stability. Therefore, it is highly desirable to design and synthesize mesoporous single-crystal microparticle materials for high-performance lithium storage, which combines microstructure and nanostructure advantages [13–22].

Hard- and soft-templating methods are the two most popular synthesis routes for constructing highly crystalline and/or single-crystal mesoporous metal oxides [23–29]. In the case of the hard-templating method, mesoporous solids are explored as templates, which enable the synthesis of a variety of highly crystalline mesoporous metal oxides. However, this method is tedious, costly and unsuitable for mass production. In contrast, the soft-templating method represents the most straightforward and feasible approach for the synthesis of mesoporous materials due to its simplicity, controllability and mass production. Many efforts have been devoted to fabricating highly crystalline mesoporous metal oxides through this route. However, the obtained compositions are usually limited to several single components [30–34]. In addition, the resultant materials are generally polycrystalline with plentiful grain boundaries and defects, which inevitably lead to negative effects in some application scenarios. Recently, multicomponent metal oxides have attracted great interest in various fields. However, to date, there is no report about the synthesis of single-crystal and stoichiometric mesoporous metal oxides with more than three components due to the following significant challenges [35,36]. (i) The drastic hydrolysis and condensation processes of different metal precursors are usually complex and unmatched with each other, thus easily resulting in macroscopic phase separation during the self-assembly process, and no mesostructures can be obtained [37–40]. (ii) The commercial surfactants are generally decomposed below 300°C, leading to the mesostructures’ collapse during the crystallization process [41]. (iii) A high temperature is required for the crystallization of multi-metal oxide to a single-crystal state, which results in the rapid growth of single-component nanocrystals independently, and eventually the breaking of mesostructures [42]. How to control the self-assembly process and relieve the contradiction between high crystallinity and porosity is still an open question in the construction of highly crystalline mesoporous metal oxides with multiple components.

Titanium-based oxides (TiO_2_, Li_4_Ti_5_O_12_, TiNb_2_O_7_, LiTi_n_O_2n+1_, etc.) have been widely used as high-performance anodes for lithium-ion batteries (LIBs) because of their high structure stability, superior safety and reversibility [43–45]. However, their high working potential and low capacity usually lead to low energy density. Notably, Li_2_TiSiO_5_, as one of the ternary metal oxides (Li_2_O-TiO_2_-SiO_2_), exhibits a two-electron (Ti^4+^/Ti^2+^ redox) conversion reaction between TiO and Li_4_SiO_4_ when being used as the anode material for LIBs. As a result, a high theoretical capacity of 308 mA h g^−1^ can be obtained [46]. More importantly, the Li_2_TiSiO_5_ also shows an appropriate and safe working potential at ∼0.28 V vs. Li^+^/Li which can not only avoid the formation of lithium dendrites but also ensure a high energy density [47,48]. These advantages make it a more promising alternative to replace commercial graphite and Li_4_Ti_5_O_12_ for LIBs. However, its low intrinsic electronic and Li^+^ conductivity of bulk form has frustrated its capacity, cycling and rate performance. Therefore, it is highly desired but challenging to construct mesoporous Li_2_TiSiO_5_ single-crystal electrodes with high-rate capability and good cycling stability.

Herein, we report the soft micelle-directed synthesis of single-crystal-like mesoporous Li_2_TiSiO_5_ via a step-crystallization strategy. To be specific, stoichiometric chelate precursor (Ti^4+^/Li^+^-citrate chelate) is first developed as a lab-made precursor. The abundant carboxyl and hydroxyl groups in the citrate can not only well coordinate Ti^4+^ and Li^+^ ions and inhibit the hydrolysis of sensitive titanium and lithium precursors but also enable successful multicomponent co-assembly into ordered mesostructures without phase separation. Subsequently, the interpenetrating carbon and SiO_2_ matrix is formed via pyrolysis, which works as rigid networks to confine the crystallization of frameworks and protect the mesostructures from collapse. Interestingly, the amorphous SiO_2_ can *in-situ* react with anisotropic Li_2_TiO_3_ to form an isotropy Li_2_TiSiO_5_ single crystal through an oriented attachment crystallization process. Meanwhile, an ultrathin carbon layer (∼2 nm) was coated on the mesopore surface. The obtained single-crystal-like mesoporous Li_2_TiSiO_5_ shows a large specific surface area (∼25 m^2^ g^−1^), uniform pore size (∼4.0 nm) and single-crystal frameworks. Notably, single-crystal-like mesoporous Li_2_TiSiO_5_ demonstrates safe working voltage potential (0.28 V vs. Li/Li^+^), a maximum reversible capacity of 393 mAh g^−1^ at 0.02 A g^−1^, superior rate capability (148 mAh g^−1^ at 5.0 A g^−1^) and outstanding long-term cycling performance (138 mAh g^−1^ at 2.0 A g^−1^ after 3000 cycles) for lithium storage.

## RESULTS AND DISCUSSION

**Figure 1.**
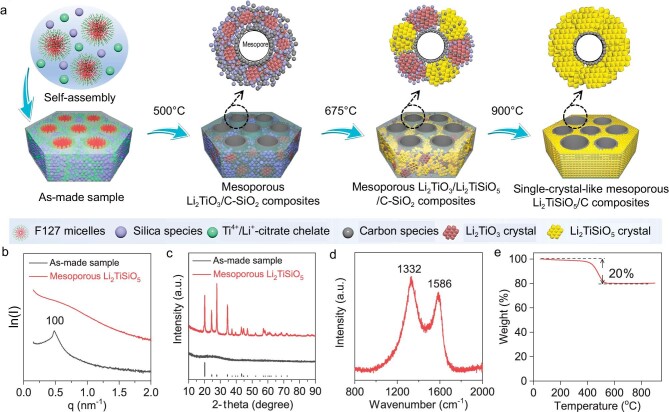
(a) Illustration of the preparation process of single-crystal-like mesoporous Li_2_TiSiO_5_ via the micelle-directed self-assembly strategy. (b) SAXS and (c) XRD patterns of the as-made sample and single-crystal-like mesoporous Li_2_TiSiO_5_, respectively. (d) Raman spectrum and (e) TGA curve of the single-crystal-like mesoporous Li_2_TiSiO_5_.

The strategy for the construction of the single-crystal-like mesoporous Li_2_TiSiO_5_ is illustrated in Fig. 1a. The stoichiometric Ti^4+^/Li^+^-citrate chelate (SCP) was prepared as the precursor for the micelle-directed self-assembly (Fig. S1). The subsequent step-crystallization process leads to the formation of single-crystal-like mesoporous Li_2_TiSiO_5_. The small-angle X-ray scattering (SAXS) pattern (Fig. 1b) of the as-made sample exhibits one clear scattering peak with *q* value of 0.485 nm^−1^, which can be attributed to the ordered mesostructures from the co-assembly between micelles and precursors. After the step-crystallization, the peak broadens and weakens, suggesting that the periodic structures of mesoporous Li_2_TiSiO_5_ have been destroyed and become disordered. The X-ray diffraction (XRD) pattern (Fig. 1c) of the as-made sample shows the typical amorphous nature without diffraction peaks of metal precursors or other products, confirming that no phase separation and crystallization occurs during the co-assembly process. After calcination at 900°C, several well-resolved diffraction peaks can be observed from the XRD pattern and indexed to the tetragonal Li_2_TiSiO_5_ structure with a *P*4*/nmm* space group (JCPDS Card No. 13-0286). The Raman spectrum (Fig. 1d) shows two bands around 1332 and 1586 cm^−1^, suggesting that *in-situ* carbonization of organic species happens during the pyrolysis process in N_2_. The carbon ratio is demonstrated to be ∼20% through the thermogravimetric analysis (TGA, Fig. 1e) result. Also, the X-ray photoelectron spectroscopy (XPS) survey spectrum shows the presence of only Ti, Si, O, Li and C elements (Fig. S2a), no other impurities can be detected. The high-resolution spectra of Ti_2p_, Si_2p_ and Li_1s_ further confirm the formation of a Li_2_TiSiO_5_ structure with Li^+^, Ti^4+^ and Si^4+^ oxidation states, and no other variable states (Fig. S2b–d) [49,50].

**Figure 2.**
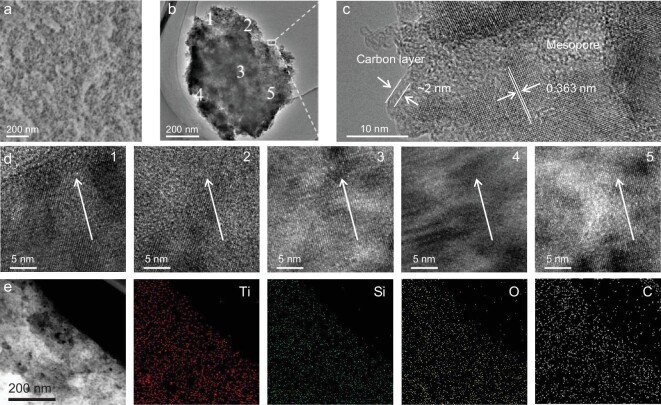
(a) SEM image, (b) TEM image and (c) HRTEM image of the mesoporous Li_2_TiSiO_5_. (d) HRTEM images of the mesoporous Li_2_TiSiO_5_ taken from region 1 to region 5 in (b). (e) STEM and elemental mapping images of the mesoporous Li_2_TiSiO_5_.

Field-emission scanning electron microscopy (SEM, Fig. 2a), transmission electron microscopy (TEM) images (Fig. 2b) and high-angle annular dark field-scanning transmission electron microscopy (HAADF-STEM) images (Fig. S3a) reveal the existence of plentiful mesopores in the single-crystal-like mesoporous Li_2_TiSiO_5_. The high-resolution TEM (HRTEM) images (Fig. 2c) and HAADF-STEM images (Fig. S3b, c) further demonstrate the presence of uniform mesopores (∼4 nm) surrounded by continuous single-crystal-like frameworks. The lattice spacing of 0.363 nm can be clearly observed from the HRTEM image, corresponding to the (101) crystalline planes of Li_2_TiSiO_5_. In addition, a carbon layer with ∼2 nm thickness can be observed on the edge of the mesoporous Li_2_TiSiO_5_, which can be attributed to the carbonization of organic species. The single-crystal-like feature can be further confirmed by the HRTEM images (Fig. 2d) taken from the different regions of the particle in Fig. 2b, which clearly show the consistent lattice orientations. In addition, the corresponding energy dispersive X-ray (EDX) spectrum (Fig. S4) and elemental mapping images (Fig. 2e) reveal that the Ti, Si, O and C elements are distributed on the framework homogeneously.

**Figure 3.**
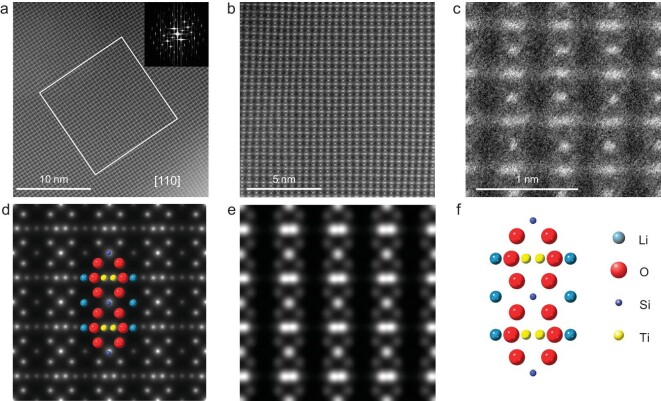
(a–c) Atomic resolution HAADF-STEM images of the single-crystal-like mesoporous Li_2_TiSiO_5_ observed along the [110] direction. (d) The projected atomic potential and (e) simulated HAADF-STEM image (with JEMS software). (f) Perspective view of the Li_2_TiSiO_5_ structure along the [110] direction.

The atomic structure of the single-crystal-like mesoporous Li_2_TiSiO_5_ was further investigated using aberration-corrected HAADF-STEM (Fig. 3). Figure 3a provides an overview of the crystal, showing a high-quality single-crystal structure. The inserted fast Fourier transformation (FFT) indicates that the sample was imaged along the [110] zone axis. The white boxed region in Fig. 3a is magnified and shown in Fig. 3b, providing details of atom locations. From the high-magnification z-contrast HAADF-STEM image in Fig. 3c, the closely neighboring pairs of titanium columns are clearly visible, with the silicon columns located in the middle of two layers of titanium. The HAADF-STEM image also reveals lighter oxygen atoms surrounding the heavier atoms, albeit with weaker contrast. This observation is consistent with both the projected atomic potential (Fig. 3d) and the simulated HAADF-STEM image using Java Electron Microscopy Simulation (JEMS) software (Fig. 3e), as well as the structure model. The STEM results clearly show the layered structure with electrochemical active TiO_6_-octahedron chains and inactive SiO_4_-tetrahedron, linked by LiO_6_-octahedron chains along the c axis, with this being thought to be the origin of the interconnected channels with large sizes of this material (Fig. 3f). As a result, the alternate arrangement of the active and inactive chains with an interconnected polyhedron network plays an important role in accommodating the volume change, and significantly contributes to crystal structure stability for lithium storage.

**Figure 4.**
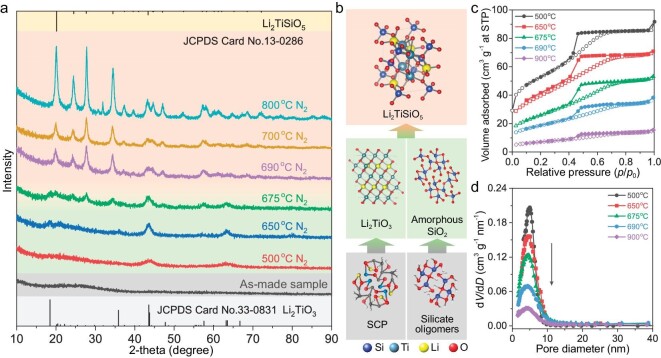
(a) XRD patterns of the samples after pyrolysis at different temperatures. (b) A schematic illustration of the transition process from precursors to Li_2_TiO_3_ and SiO_2_ and then to Li_2_TiSiO_5_. (c) N_2_ sorption isotherms and (d) pore size distribution curves of samples after pyrolysis at different temperatures, respectively.

The structural evolution process of the single-crystal-like mesoporous Li_2_TiSiO_5_ with increased pyrolysis temperature was investigated to understand the formation mechanism. XRD patterns (Fig. 4a) show that the frameworks can be crystallized into Li_2_TiO_3_ phase at 500°C and then turn to the mixed phases of Li_2_TiO_3_ and Li_2_TiSiO_5_ phase at 675°C. Diffraction peaks of pure Li_2_TiSiO_5_ phase can be observed with the temperature >690°C. Then, the peak intensity of pure Li_2_TiSiO_5_ phase increases with the rise of pyrolysis temperature, demonstrating the increased crystallinity and particle size. The gradually increasing average grain size was also estimated by the Scherrer equation to support the enhanced crystallinity with increasing annealing temperature (Fig. S5). These results reveal that the titanium and lithium precursors can be transformed to crystalline Li_2_TiO_3_ phase at a low pyrolysis temperature, which can then *in-situ* react with the SiO_2_ matrix at solid phase into Li_2_TiSiO_5_ at a higher temperature (Fig. 4b). That can be further confirmed by the nitrogen sorption analysis (Fig. 4c). All samples show typical type-IV isotherms with a capillary condensation step in the P/P_0_ of 0.42−0.65, suggesting the existence of mesopores. Their corresponding Brunauer–Emmett–Teller (BET) surface areas and pore volume decrease with the increased pyrolysis temperatures, demonstrating the gradual increased crystallinity and density of the frameworks (Table 1). Notably, the pore-size distribution curves (Fig. 4d) display slightly decreased pore sizes but greatly decreased pore volume with an increase in the pyrolysis temperature, indicating that the crystal sizes of the frameworks are almost unchanged, nevertheless, the overall density dramatically increases.

**Table 1.** Physicochemical properties of the samples after pyrolysis at different temperatures.

Pyrolysis temperature (°C)	BET surface area (m^2^ g^−1^)	Pore size (nm)	Pore volume (cm^3^ g^−1^)	Crystal phase	Crystalline state
500	172	4.8	0.113	Li_2_TiO_3_	Polycrystals
650	131	4.6	0.087	Li_2_TiO_3_	Polycrystals
675	90	4.5	0.065	Li_2_TiO_3_/Li_2_TiSiO_5_	Polycrystals
690	65	4.3	0.045	Li_2_TiSiO_5_	Polycrystals
900	25	4.0	0.024	Li_2_TiSiO_5_	Single crystals

**Figure 5.**
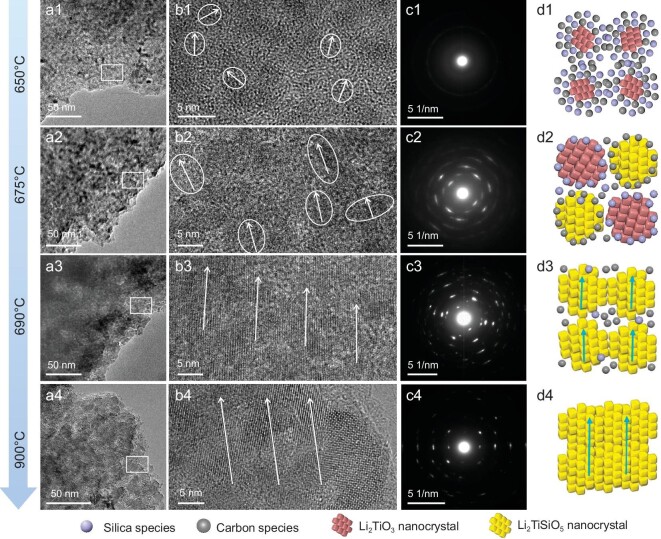
(a1–a4) TEM images, (b1–b4) HRTEM images, (c1–c4) the corresponding SAED patterns and (d1–d4) schematic illustration of the structural evolution process of the mesoporous Li_2_TiSiO_5_ prepared via the step-crystallization route. The stoichiometric metal precursors ensure the formation of pure Li_2_TiO_3_ and Li_2_TiSiO_5_ without impurities.

Figure 5a and b display the TEM and HRTEM images of the samples after calcination at different temperatures. TEM images of the sample obtained after pyrolysis at 650°C show that the small nanocrystals of Li_2_TiO_3_ are randomly dispersed in the pore walls and wrapped by an amorphous matrix, which can gradually grow into bigger nanocrystals of Li_2_TiSiO_5_ with aligned orientations at 675°C. Further increasing the calcination temperature makes the nanocrystals fuse together with almost the same lattice orientations and better crystallinity. In addition, the mesoporous structures and surface supporting carbon layer are not changed so much and can be well retained. This crystallization process can be attributed to an oriented arrangement-based crystal growth mechanism [51–53]. This growth mechanism concludes that the electrostatic field surrounding the nanocrystals can provide the orienting force, which promotes the oriented attachment that occurs on the high-surface-energy faces between two crystalline particles under the confinement of a carbon and SiO_2_ matrix. Then, these two nanocrystals fuse into one large nanocrystal to reduce the surface energy. As a result, single-crystal-like Li_2_TiSiO_5_ can be obtained after consecutive growth and fusion processes. The selected area electron diffraction (SAED) patterns (Fig. 5c) further demonstrate the transition process of the frameworks from spotty diffraction rings of polycrystalline nature to single-crystal-like diffraction spots. In addition, the retention of mesopores in the crystal growth process can further demonstrate that the crystallization process is mild and controllable.

Control experiments were conducted to further understand the formation mechanism of single-crystal-like mesoporous Li_2_TiSiO_5_. First, no diffraction peaks and mesostructures can be observed from the SAXS pattern and TEM image without the presence of soft micelles, but single-crystal Li_2_TiSiO_5_ particles can still be obtained, further confirming that the solid reaction would happen between the mixed precursors at a high temperature (Fig. S6). Second, when using commercial titanium alkoxides and LiNO_3_ as the precursors, the SAXS pattern shows that disordered mesostructures can be obtained after multicomponent co-assembly, but disappear after calcination at 500°C, suggesting instability (Fig. S7a). The XRD patterns reveal that typical diffraction peaks of the LiNO_3_ phase can be detected in the as-made sample and several scattering peaks of the Li_2_TiO_3_ phase are observed after calcination at 500°C (Fig. S7b). When being calcined at 900°C, the HRTEM image and the corresponding SAED pattern demonstrate the formation of a pure Li_2_TiSiO_5_ single crystal without mesopores, which can be further confirmed by the XRD and N_2_ adsorption-desorption results (Fig. S8). Third, no silica source in the synthetic system was also conducted. It was found that the mesoporous structures can be obtained at a low calcination temperature, but degraded at 900°C due to the aggregate and growth of Li_2_TiO_3_ particles (Fig. S9), in accordance with the XRD and N_2_ adsorption results (Fig. S10). Besides, no single crystal can be observed in this case.

Based on the above observations, a step-crystallization mechanism was proposed for the formation of the single-crystal-like mesoporous Li_2_TiSiO_5_ (Fig. 5d). Herein, stoichiometric chelate precursor was first prepared, which is the key for the following co-assembly. The carboxyl groups of citric acid can effectively coordinate Ti^4+^ and Li^+^ ions, giving them very stable atomic dispersion. Therefore, the uncontrollable hydrolysis process can be avoided in the synthetic process. At the same time, the abundant hydroxyl groups enable the strong hydrogen-bond interaction with the soft micelles and silicate oligomers. As a result, the soft micelles can direct the modular co-assembly into ordered mesostructures with homogeneously mixed frameworks at the atomic scale. When subjected to calcination, the soft micelles are burned off first and leave the mesopores. Meanwhile, the pure Li_2_TiO_3_ nanocrystals begin to nucleate and grow in the framework with the increase in pyrolysis temperature. Interestingly, the citrate ligands and silicate oligomers can be *in-situ* converted to the rigid carbon and SiO_2_ networks during this step, which well confines the growth of Li_2_TiO_3_ nanocrystals and protects the mesoporous structure from collapse. Further increasing the temperature triggers the second step crystallization. Tiny Li_2_TiSiO_5_ nanocrystals are formed by the solid reaction between stoichiometric SiO_2_ and Li_2_TiO_3_. The amorphous SiO_2_ matrix not only acts as a rigid template to confine the growth, but also works as a precursor for Li_2_TiSiO_5_. More importantly, the oriented arrangement of Li_2_TiSiO_5_ nanocrystals would be dynamically changed from anisotropic to isotropic due to the confinement effect of mesostructures, which ensures oriented attachment-based crystallization, leading to the formation of single-crystal-like Li_2_TiSiO_5_ without structure collapse. The accurate stoichiometric ratio of Li/Ti/Si ensures the formation of pure Li_2_TiO_3_ and then Li_2_TiSiO_5_ in the synthesis process without impurities.

**Figure 6.**
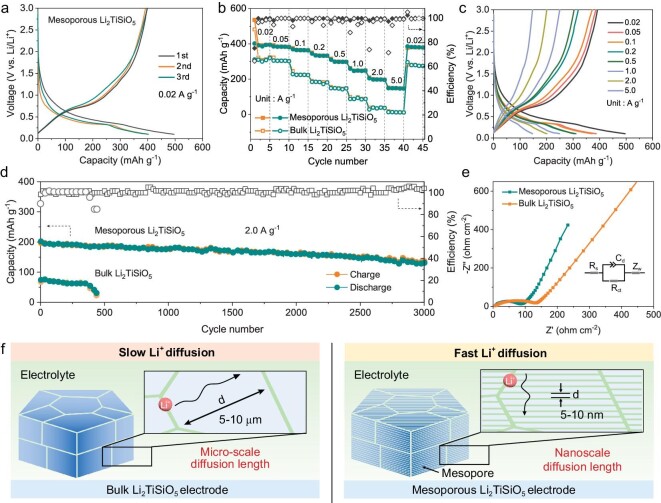
(a) Charge-discharge curves of the single-crystal-like mesoporous Li_2_TiSiO_5_ for the initial three cycles. (b) Rate performance of the mesoporous and bulk Li_2_TiSiO_5_. (c) Charge-discharge curves at different current densities of the single-crystal-like mesoporous Li_2_TiSiO_5_. (d) Cycling performance of the mesoporous and bulk Li_2_TiSiO_5_ at a current density of 2.0 A g^−1^. (e) Nyquist plots of the single-crystal-like mesoporous Li_2_TiSiO_5_ sample. (f) Schematic diagram of the Li^+^ diffusion mechanism in bulk and mesoporous Li_2_TiSiO_5_ electrodes.

The lithium storage performance of the single-crystal-like mesoporous Li_2_TiSiO_5_, bulk Li_2_TiSiO_5_ and Li_2_TiSiO_5_ sample obtained after pyrolysis at 690°C were investigated. The cyclic voltammetry (CV) curves revealed that the reduction peaks of single-crystal-like mesoporous Li_2_TiSiO_5_ were at ∼0.28 V during the discharge process, consistent with previous reports, while weaker peaks at ∼0.1 V could be attributed to the conversion reaction from Li_2+_*_x_*TiSiO_5_ to Li_4_SiO_4_ and TiO, and a solid-solution reaction of TiO [49,54]. In addition, a broadening reduction peak at ∼0.8 V can be observed at the initial cycle and then disappears in subsequent cycles, demonstrating the formation of an irreversible solid electrolyte interphase (SEI) (Fig. S11). The charge-discharge curves of the first, second and third cycles were recorded at 0.02 A g^−1^ (Fig. 6a). A clear plateau at 0.28 V and two sloping curves below and above the plateau can be observed, showing that three reaction steps are included in the lithium storage process of the single-crystal-like mesoporous Li_2_TiSiO_5_ anode. More importantly, it delivered initial discharge and charge capacities of 496 and 393 mAh g^−1^, with an initial Coulombic efficiency (CE) of 79.2%.

The rate performance of single-crystal-like mesoporous Li_2_TiSiO_5_ and bulk Li_2_TiSiO_5_ were further measured systematically. Single-crystal-like mesoporous Li_2_TiSiO_5_ maintains high reversible capacities of 393, 384, 364, 333, 298, 247, 198 and 148 mAh g^−1^ at 0.02, 0.05, 0.1, 0.2, 0.5, 1.0, 2.0 and 5.0 A g^−1^, respectively (Fig. 6b). Bulk Li_2_TiSiO_5_ only maintains 13 mAh g^−1^ at 5.0 A g^−1^. Also, the capacity of single-crystal-like mesoporous Li_2_TiSiO_5_ can quickly return to 380 mAh g^−1^ when the current density is turned back to 0.02 A g^−1^, showing excellent rate performance and stability. Charge and discharge curves of single-crystal-like mesoporous Li_2_TiSiO_5_ at different current densities exhibit high symmetry implying small polarization (Fig. 6c). Bulk Li_2_TiSiO_5_ delivers discharge platforms with significant changes and polarization (Fig. S12). The cycling stability of single-crystal-like mesoporous Li_2_TiSiO_5_ was further measured systematically. It maintains a high reversible capacity of 201 mAh g^−1^ after 1400 cycles at 0.2 A g^−1^. Meanwhile, bulk Li_2_TiSiO_5_ maintains a negligible reversible capacity. The CE of single-crystal-like mesoporous Li_2_TiSiO_5_ is closely kept at ∼100%, compared to that of bulk Li_2_TiSiO_5_ with obvious fluctuations (Fig. S13). At higher current density, single-crystal-like mesoporous Li_2_TiSiO_5_ still delivers excellent cycling stability, with a reversible capacity of 138 mAh g^−1^ after 3000 cycles at 2.0 A g^−1^ (0.01% decay per cycle), however, the capacity of the bulk Li_2_TiSiO_5_ decreases sharply after 400 cycles (Fig. 6d). Charge and discharge curves of single-crystal-like mesoporous Li_2_TiSiO_5_ at different cycles reveal excellent voltage stability performance during repeated lithiation/de-lithiation processes (Fig. S14).

The lithium storage performance of the mesoporous Li_2_TiSiO_5_ sample obtained after pyrolysis at 690°C was also measured to support the influence of different Li_2_TiSiO_5_ crystallinities on electrochemical performance. There are no obvious reaction peaks around 0.28 V in the CV curves, indicating that weak crystallinity is not conducive to the discharge plateaus, but tends to form discharge curves with sloping characteristics (Fig. S15a). The mesoporous Li_2_TiSiO_5_ sample obtained after pyrolysis at 690°C delivered initial discharge and charge capacities of 582 and 373 mAh g^−1^ at 0.02 A g^−1^, with an initial CE of 64.1% (Fig. S15b). In the second cycle, it showed a reversible discharge capacity of 362 mAh g^−1^, however, a reversible capacity of 168 mAh g^−1^ at a high current density of 2 A g^−1^ was achieved, which may be due to the poor conductivity of carbon coating at lower calcination temperatures (Fig. S15c). The discharge profiles of the Li_2_TiSiO_5_ sample with weaker crystallinity measured at 0.02 A g^−1^ show two plateaus at ∼0.28 V and ∼0.15 V, but these plateaus remain irresolvable at high current densities (Fig. S15d), indicating poor kinetics and platform retention. In addition, single-crystal-like mesoporous Li_2_TiSiO_5_ has a significantly higher capacity and superior rate and cycling performance than reported Li_2_TiSiO_5_ materials (Table S1). Nyquist plots of the single-crystal-like mesoporous Li_2_TiSiO_5_ electrode present a depressed semicircle with a smaller diameter in the moderate frequency region, and a straight line with a higher slope in the low-frequency region, than bulk Li_2_TiSiO_5_, demonstrating that the single-crystal-like mesoporous Li_2_TiSiO_5_ possesses lower charge-transfer resistance than bulk Li_2_TiSiO_5_ (Fig. 6e).

The maximum lithium storage capacity, superior rate capability and outstanding cycling performance of the single-crystal-like mesoporous Li_2_TiSiO_5_ can be attributed to its unique nanostructures and single-crystal nature. The uniform mesoporous structures facilitate the transport of electrolytes [55]. The high surface areas provide sufficient contacts between electrode and electrolyte, thus leading to rapid electrochemical reactions and high lithium storage capacities. In addition, the mesopores can greatly facilitate fast Li^+^ diffusion through short nanoscale diffusion lengths (5–10 nm), which is beneficial for improving rate capability and cycling performance. Meanwhile, the existence of conductive carbon networks on the pore surface and single-crystal features is beneficial for fast electron transfer through the electrode. However, due to the micron-scale diffusion lengths (5-10 μm), bulk Li_2_TiSiO_5_ exhibits slow Li^+^ diffusion, resulting in poor rate and cycling performance (Fig. 6f).

## CONCLUSION

In summary, we have demonstrated soft micelle self-assembly to prepare single-crystal-like mesoporous Li_2_TiSiO_5_, via a step-crystallization route, for high-performance LIBs. Here, the assembly and crystallization processes can be decoupled and well controlled for multicomponent co-assembly into ordered mesostructures. The homogeneously mixed nature of stoichiometric chelate precursors at the atomic-scale level enables the precise regulation of the crystallization process. The citrate and silicates can be converted to rigid carbon and SiO_2_ networks, which simultaneously confine the crystallization of frameworks without structure collapse. An oriented attachment crystallization process from anisotropic to isotropic single crystal happens under the confinement effect, leading to well-defined mesoporous structures with single-crystal-like frameworks. As a result, the single-crystal-like mesoporous Li_2_TiSiO_5_ exhibits a safe working potential (∼0.28 V vs. Li/Li^+^), maximum lithium storage of 393 mAh g^−1^ at 0.02 A g^−1^, superior rate capability (148 mAh g^−1^ at 5.0 A g^−1^) and outstanding long-term cycling performance (138 mAh g^−1^ at 2.0 A g^−1^ after 3000 cycles) due to fast Li^+^ diffusion caused by mesochannels, which correspond to nanosized crystal frameworks and short diffusion lengths (5–10 nm). The atomic structure of Li_2_TiSiO_5_ crystals was also characterized and obtained, which helps us to understand the performance improvement mechanism of single-crystal-like mesoporous Li_2_TiSiO_5_. We envisage that such coordination-regulated self-assembly combined with a step-crystallization strategy affords us a new methodology to design and synthesize highly crystalline and stoichiometric mesoporous multicomponent metal oxides.

## Supplementary Material

nwae054_Supplemental_File
