# Supplementary material for: Micelle-directed self-assembly of single-crystal-like mesoporous stoichiometric oxides for high-performance lithium storage
Source: Natl Sci Rev. 2024 Feb 6;11(4):nwae054. doi: 10.1093/nsr/nwae054 (PMC10972673; doi:10.1093/nsr/nwae054)
Supplement: nwae054_Supplemental_File [file nwae054_supplemental_file.pdf]

Supporting Information

Micelles directed self-assembly to single-crystal-like mesoporous stoichiometric oxides for high-performance lithium storage

Yanhua Wan,[1,†] Changyao Wang,[1,†] Xingmiao Zhang,[1] Yang Yin,[2] Mengmeng Liu,[1] Bing Ma,[1] Linlin Duan,[1] Yuzhu Ma,[1] Wei Zhang,[1] Changlin Zheng,[2] Dongliang Chao,[1] Fei Wang,[1] Yongyao Xia[1] and Wei Li[*,1]

[1]Department of Chemistry, Laboratory of Advanced Materials, Shanghai Key Lab of Molecular Catalysis and Innovative Materials, State Key Laboratory of Molecular Engineering of Polymers, College of Chemistry and Materials Science, Fudan University, Shanghai 200433, P. R. China

[2]State Key Laboratory of Surface Physics and Department of Physics, Fudan University, Shanghai 200433, P. R. China

[†]Yanhua Wan, Changyao Wang contributed equally.

[*]**Corresponding authors.** E-mail: weilichem@fudan.edu.cn

Experimental section

Chemicals. Titanium isopropoxide ($Ti(OCH(CH_3)_2)_4$, TIPO, ⩾ 97 %) was purchased from Fluka. Poly(ethylene oxide)-block-poly(propylene oxide)-block-poly(ethylene oxide) triblock copolymer Pluronic F127 (Mw = 12600, PEO_{106}-PPO_{70}-PEO_{106}) was purchased from Aldrich. Tetraethyl orthosilicate ($Si(OC_2H_5)_4$, TEOS, ≥96 wt%), citric acid monohydrate ($C_6H_8O_7 \cdot H_2O$, AR), hydrogen chloride (HCl, AR), and ethanol (AR) were purchased from Sino−Pharm Chemical Reagent Co. Ltd. Lithium nitrate ($LiNO_3$, AR) was purchased from Aladdin. All the chemicals were used without further purification. Deionized water was used for all experiments.

Synthesis of Stoichiometric Ti^{4+}/Li^{+}-Citrate Chelate Precursor. The stoichiometric Ti^{4+}/Li^{+}-citrate chelate precursor (SCP) was synthesized according to our previous method.[1] For a typical preparation, citric acid (21.0 g, 100 mmol) was dissolved in 100 mL of ethanol (solution A), titanium isoproxide (28.4 g, 100 mmol) was dissolved in 50 mL of ethanol (solution B), and lithium nitrate (13.8 g, 200 mmol) was dissolved in 50 mL of ethanol (solution C). Solution A was dropped into solution B slowly and heated with stirring at 50 °C for 3 h (solution D). Then, solution C was dropped into solution D and heated with stirring for another 3 h. Ethanol was distilled off under vacuum at 45 °C. The obtained gel was then re-dissolved in water to obtain a 1.0 M stoichiometric Ti^{4+}/Li^{+}-Citrate chelate precursor (SCP) solution.

Synthesis of Single-Crystal-Like Mesoporous Li_2TiSiO_5. The single-crystal-like mesoporous Li_2TiSiO_5 was synthesized *via* the micelles-directed step-crystallization strategy. In a typical synthesis, 1.5 g of triblock copolymer Pluronic F127, 0.83 g of TEOS, and 4.0 mL of 1.0 M SCP solution were dissolved in an ethanol/water mixture (10 mL, 4:1 v/v). After stirring for 3 h at room temperature, the obtained light yellow homogenous solution was poured into Petri dishes to evaporate the solvents at 40 °C for 8 h, followed by sequential solidifying the samples at 100 °C for 20 h in an oven to remove the solvents completely, leading to the formation of the as-made F127/Ti^{4+}/Si^{4+}/Li^{+}/Citrate composites (denoted as as-made sample). Then the as-made

sample was pyrolyzed at 900 °C for 3 h with a ramp of 2 °C min^{-1} in N_2 atmospheres, resulting in the mesoporous Li_2TiSiO_5.

Characterization. Small-angle X-ray scattering (SAXS) measurements were taken on a Nanostar U small-angle X-ray scattering system (Bruker, Germany) using Cu Kα radiation (40 kV, 35 mA). X-ray diffraction (XRD) patterns were obtained from a Bruker D4 diffractometer within 2θ range from 10 to 90°, by using a Cu Kα radiation source (40 kV, 40 mA). Nitrogen adsorption−desorption isotherms were measured at 77 K with a Micromeritics Tristar 2420 analyzer (USA). Before measurements, the samples were degassed in a vacuum at 190 °C for 10 h. The Brunauer−Emmett−Teller (BET) method was utilized to calculate the specific surface areas. By using the Barrett−Joyner−Halenda (BJH) model, the pore volumes and pore size distributions were derived from the adsorption branches of isotherms, and the total pore volumes were estimated from the adsorbed amount at a relative pressure P/P_0 of 0.992. Transmission electron microscopy (TEM) measurements were conducted on o JEM-2100 F microscope (JEOL, Japan) operated at 200 kV. The atomic resolution HAADF-STEM imaging was conducted with a field-emission transmission electron microscope (Themis Z, Thermo Fisher Scientific) equipped with double aberration correctors (SCORR probe corrector and CETCOR image corrector, CEOS GmbH). The microscope was operated at 300 kV. For STEM-HAADF imaging, the semi-convergent angle of the probe forming lens was set to 21.4 mrad, and the semi-collection angle of the annular dark field detector is from 79 mrad to 200 mrad. The samples for the TEM measurements were suspended in ethanol and supported onto a holey carbon film on a Cu grid. Field-emission scanning electron microscopy (FESEM) images were taken on a Hitachi S-4800 microscope. The dried samples were directly used for the observation without any treatment. X-ray photoelectron spectroscopy (XPS) was recorded on an AXIS ULTRA DLD XPS System with MONO Al source (Shimadzu Corp). Photoelectron spectrometer was recorded by using monochromatic Al KR radiation under vacuum at 5×10^{-9} Pa. All calibrations were referenced to the surface adventitious carbon (C1s = 284.6 eV). Elemental analysis experiments were carried out using an Elementar Vario EL III microanalyzer.

Raman spectra were recorded with a Dilor LabRam-1 B microscopic Raman spectrometer, using a He−Ne laser with an excitation wavelength of 632.8 nm.

Cell assembly and electrochemical measurements. The electrodes were fabricated by mixing the active material, conductive agent Super P, and polymer binder (Carboxymethylcellulose sodium, CMC) at a weight ratio of 8:1:1. This mixture was dispersed in deionized water to form a slurry and then cast on the Cu foil. Then, the electrodes were dried at 80 °C in a vacuum oven overnight. The average mass loading was about 1.5 mg cm^{-2}. 2025 coin-type lithium-ion cells were assembled in an argon-filled glovebox with moisture and oxygen concentration below 1.0 ppm for electrochemical characterization. 1.0 M $LiPF_6$ in a 1:1:1 volume ratio of ethyl methyl carbonate (EMC), dimethyl carbonate (DMC), and ethylene carbonate (EC) was used as the electrolyte. Li metal foils were used as counter electrodes for electrochemical measurement. Cyclic voltammograms (CV) were obtained using a CHI660e electrochemical workstation at a sweep rate of 0.1 mV s^{-1} within a range from 0.1−3.0 V. The charge/discharge tests were performed on NEWARE Battery Test System (CT-4008, Shenzhen, China) within a voltage range of 0.1−3.0 V vs. Li/Li^+ at room temperature. For the high-rate testing, the charge/discharge current gradually increased from 0.02 to 0.05, 0.1, 0.2, 0.5, 1.0, 2.0 and 5.0 A g^{-1}, then decreased to 0.02 A g^{-1}. The cycle performance at high current density (0.2 and 2.0 A g^{-1}) was activated at 0.02 A g^{-1} for five cycles. Electrochemical impedance spectroscopy (EIS) spectra were collected from 100 kHz to 100 MHz at room temperature (25 °C).

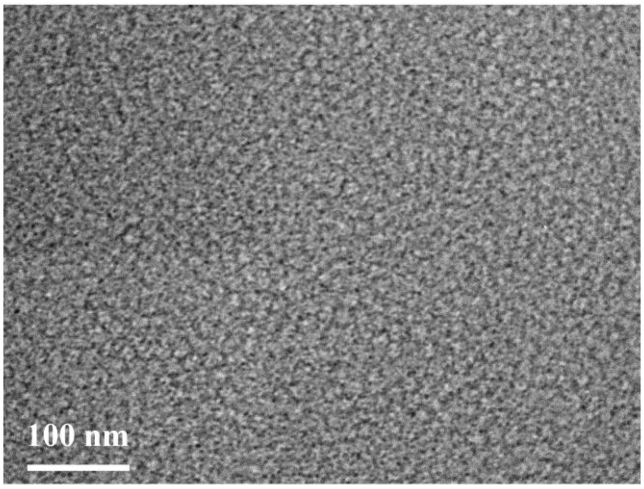

Figure S1. TEM image of the spherical F127/SCP/SiO_2 composite micelles.

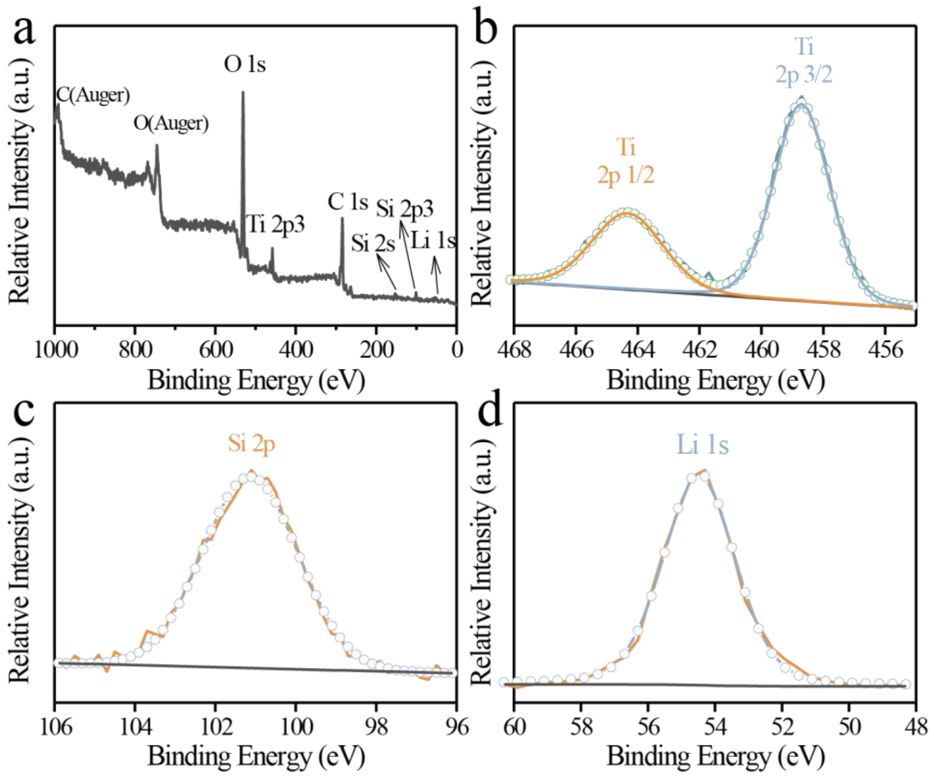

Figure S2. (a) The XPS survey spectrum and (b-d) XPS core-level spectra of Ti 2p, Si 2p, and Li 1s of the mesoporous Li_2TiSiO_5 prepared by the micelles-directed self-assembly and step-crystallization strategy after the pyrolysis at 900 °C in N_2.

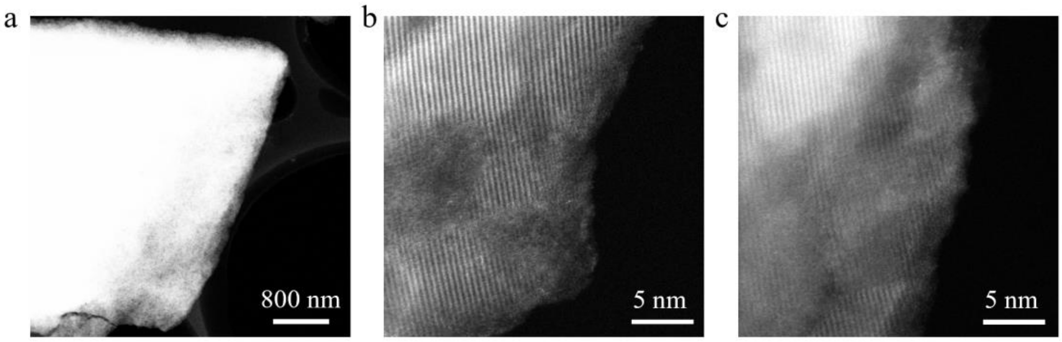

Figure S3. (a-c) HAADF-STEM images of single-crystal-like mesoporous Li_2TiSiO_5.

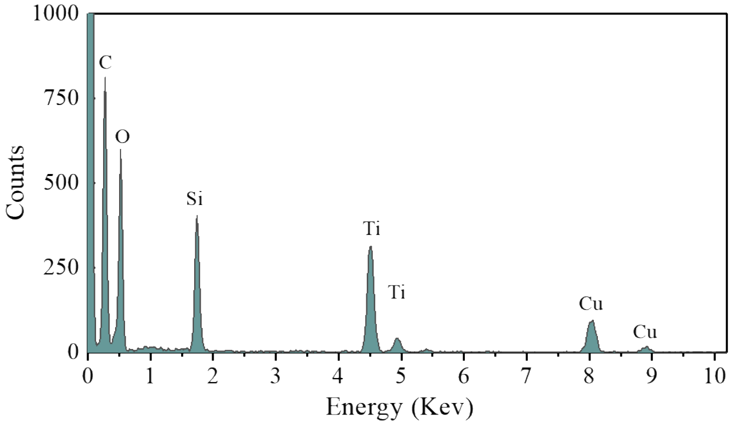

Figure S4. The EDX spectrum of the mesoporous Li_2TiSiO_5 obtained after the pyrolysis at 900 °C in N_2.

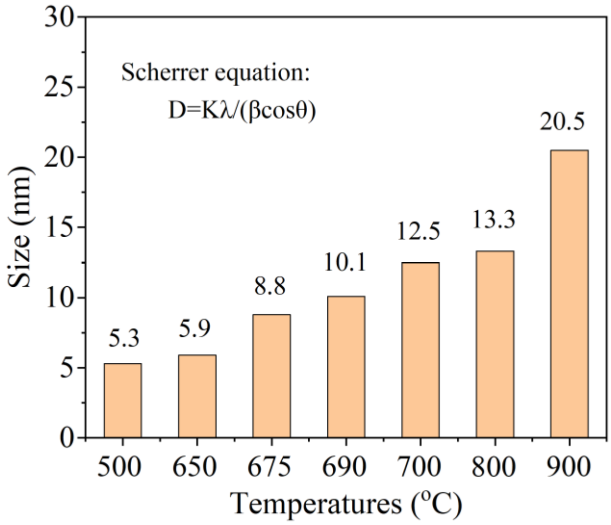

Figure S5. Size distribution of the samples after pyrolysis at different temperatures.

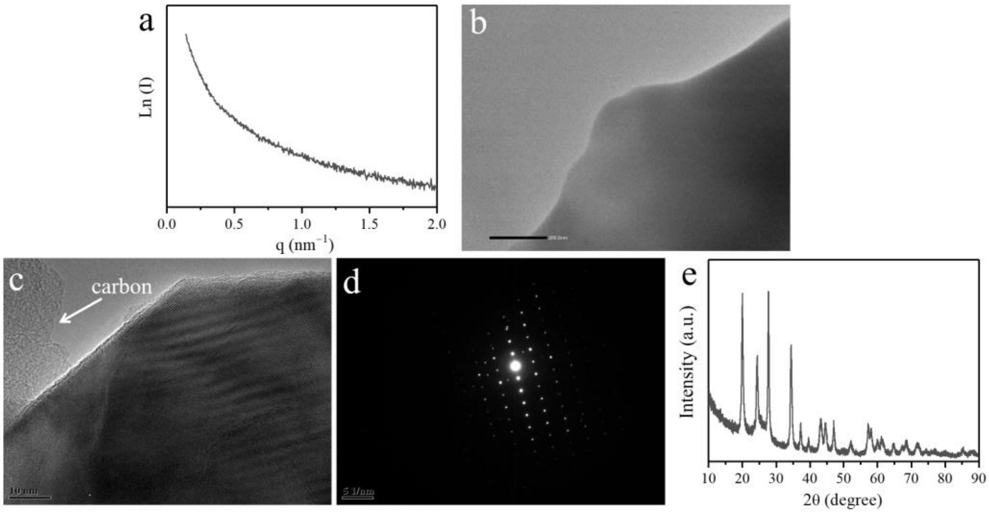

Figure S6. SAXS pattern (a), TEM image (b) of the as-made sample. TEM image (c) and corresponding SAED pattern (d), and XRD pattern (e) of bulk Li2TiSiO5 after pyrolysis at 900 ℃ in N_2 prepared without the addition of Pluronic F127 in the synthesis.

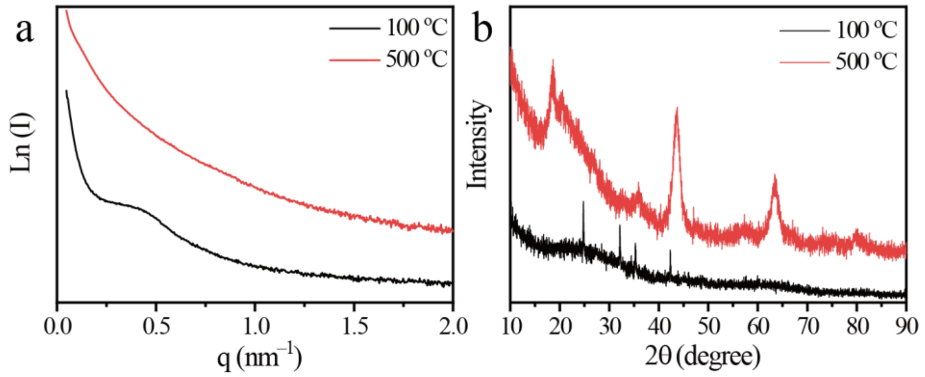

Figure S7. SAXS (a) and XRD (b) patterns of samples obtained after heat-treatment at 100 and 500 ℃ prepared by using TIPO and $LiNO_3$ as precursors, respectively.

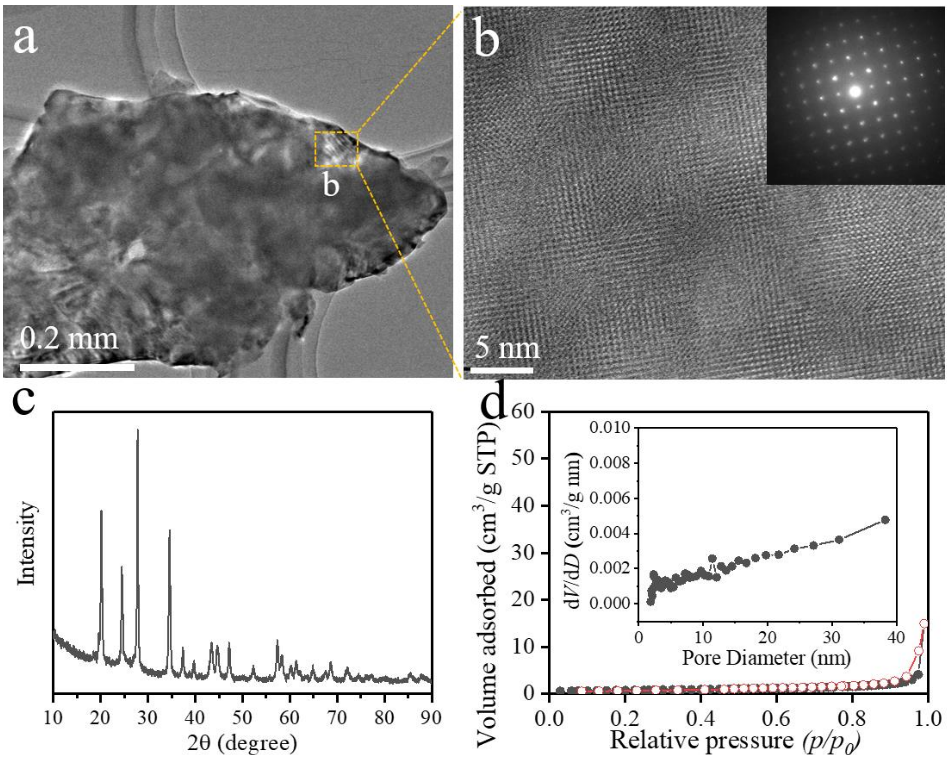

Figure S8. TEM image (a), HRTEM image (b), XRD pattern (c), Nitrogen-sorption isotherms and pore size distribution curves (b) of the sample obtained after pyrolysis at 900 ℃ in N_2 prepared by using TIPO and $LiNO_3$ as precursors, respectively.

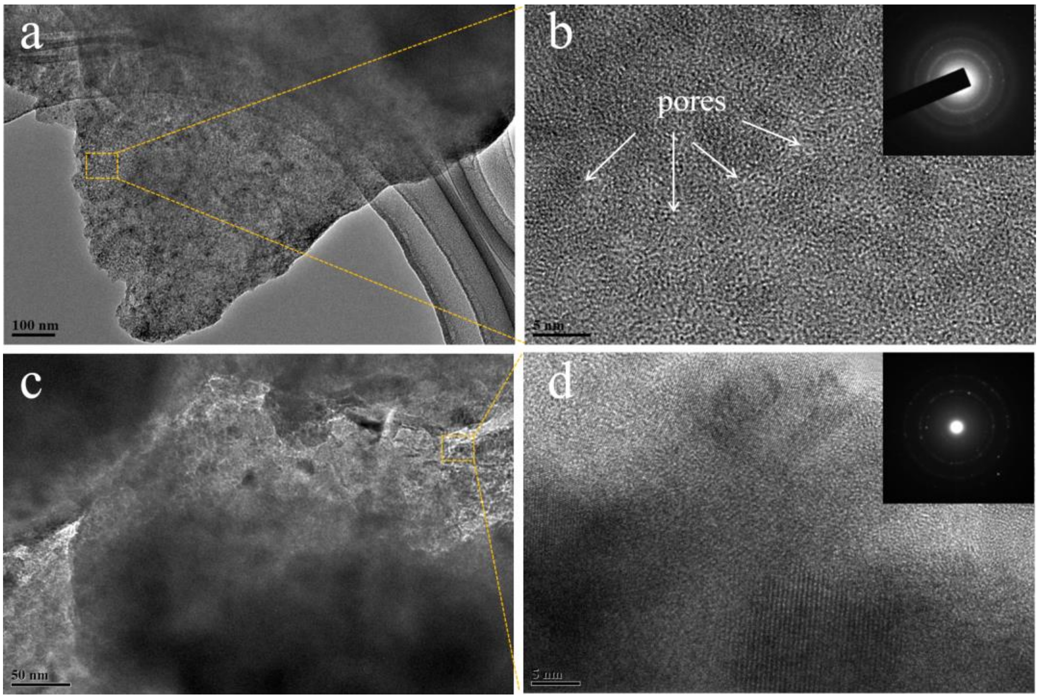

Figure S9. TEM, HRTEM, and corresponding SAED pattern of the sample obtained after pyrolysis at 500 ℃ (a, b) and 900 ℃ (c, d) in N_2, respectively, prepared without the addition of silica precursors in the synthesis system.

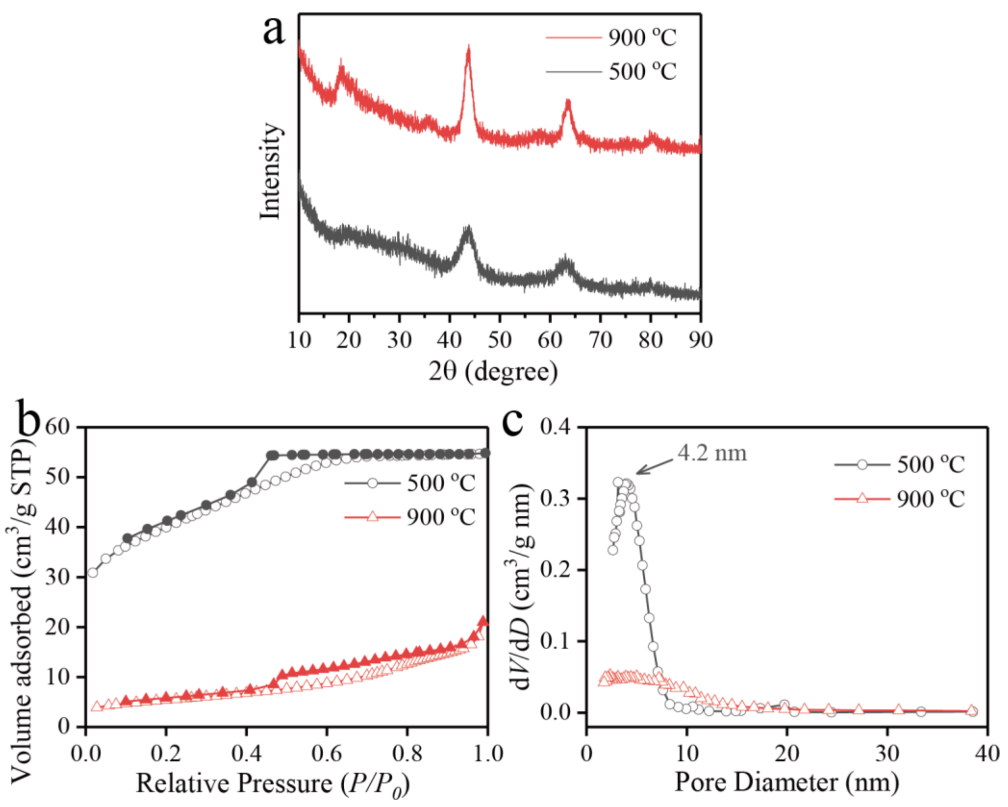

Figure S10. XRD pattern (a), nitrogen-sorption isotherms (b), and the corresponding pore size distribution curves (c) of the sample obtained after pyrolysis at 500 and 900 ℃ in N_2, respectively, prepared without the addition of silica precursors in the synthesis system.

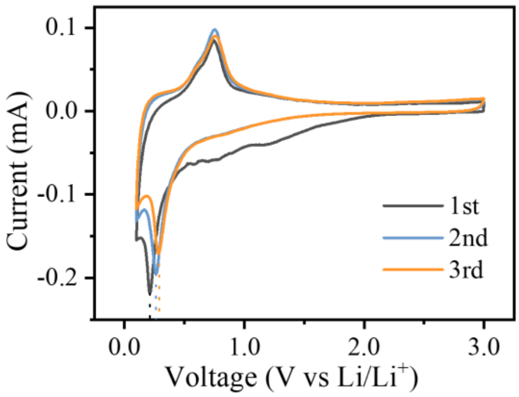

Figure S11. CV curves of the mesoporous Li_2TiSiO_5 for the initial three cycles at a scan rate of 0.1 mV s^{-1}.

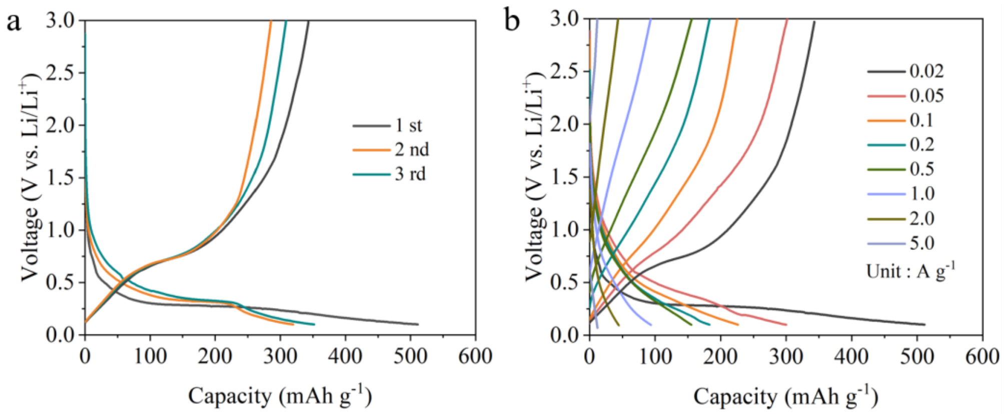

Figure S12. Charge-discharge curves of the bulk Li_2TiSiO_5 at (a) initial three cycles and (b) different current densities.

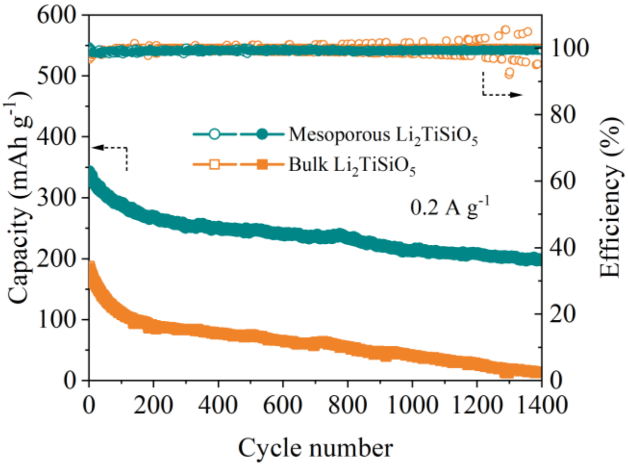

Figure S13. Charge-discharge curves of the bulk Li_2TiSiO_5 at (a) initial three cycles and (b) different current densities.

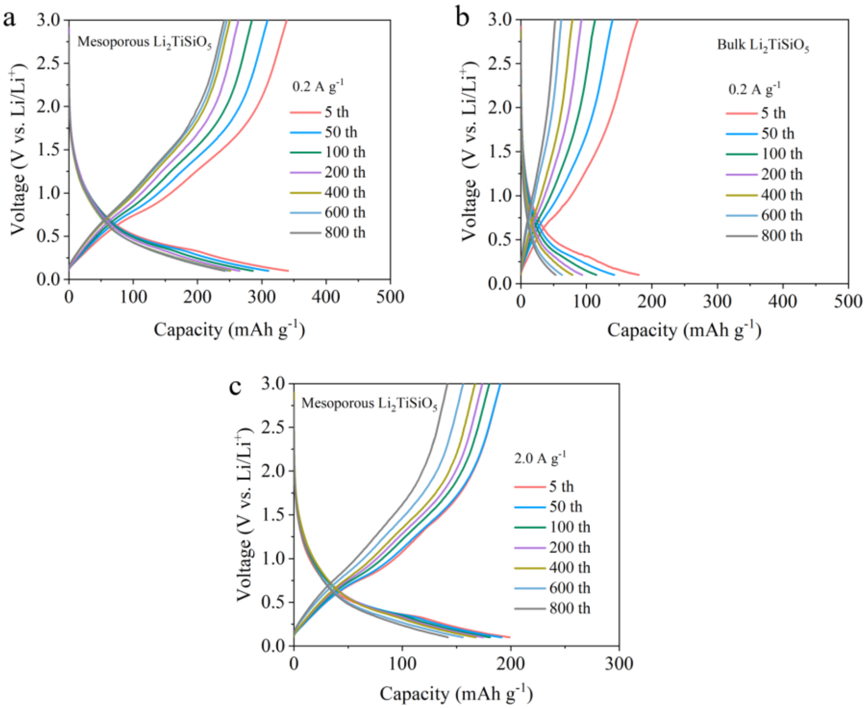

Figure S14. Charge-discharge curves of different cycles at 0.2 A g^{-1} of (a) mesoporous Li_2TiSiO_5 and (b) bulk Li_2TiSiO_5. Charge-discharge curves of different cycles at 2.0 A g^{-1} of (c) mesoporous Li_2TiSiO_5.

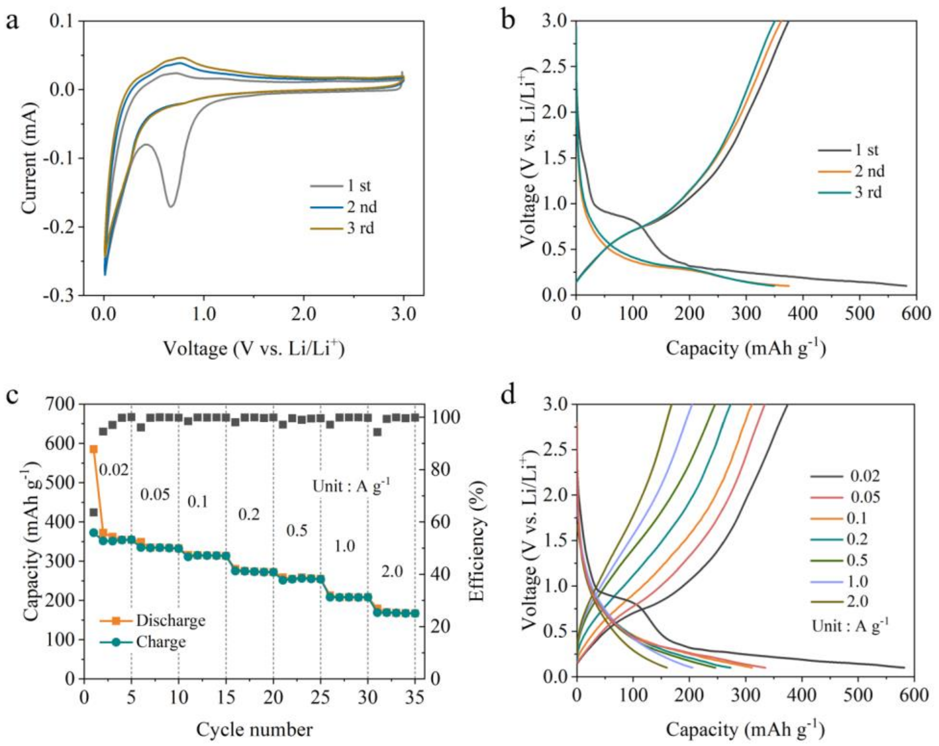

Figure S15. (a) CV curves, (b) Charge-discharge curves, (c) Rate performance, and (d) Charge-discharge curves at different current densities of the Li_2TiSiO_5 sample obtained after pyrolysis at 690 ℃.

Table S1. The lithium storage performance of the reported Li_2TiSiO_5 materials.

Material	Potential range (V vs Li/Li^+)	Specific capacity (mAh g^{-1}/A g^{-1})	Rate capacity (mAh g^{-1}/A g^{-1})	Cycling stability (mAh g^{-1}/Cycles/A g^{-1})	Ref.
Fibrous Li_2TiSiO_5/C	0.1–3.0	265.9/0.1	151.2/5.0	~180/1000/0.5<br>~160/2000/5	[2]
TiO_2- Li_2TiSiO_5	0.1–3.0	325/0.1	150/1.0	300/100/0.1<br>72/10000/2.0	[3]
Li_2TiSiO_5/N-C	0.01–3.0	332.6/0.05	108.3/4.0	240.6/500/1.0	[4]
C@ Li_2TiSiO_5/CNT	0–3.0	430/~0.16	125/3.15	~125/1500/3.15	[5]
3DC@ Li_2TiSiO_5	0–3.0	280/0.2	80/4.0	~80/3000/2.0	[6]
Li_2TiSiO_5	0–3.0	~300/0.02	~200/1.0	~160/1000/0.5	[7]
G- Li_2TiSiO_5	0.1–3.0	242.3/0.03	100/4.5	200/50/0.3	[8]
**Single-crystal-like mesoporous Li_2TiSiO_5**	**0.1–3.0**	**393/0.02**	**148/5.0**	**201/1400/0.2**<br>**138/3000/2.0**	**This work**

References

[1] Wang C, Wan X and Duan L *et al*. Molecular Design Strategy for Ordered Mesoporous Stoichiometric Metal Oxide. *Angew Chem Int Ed* 2019; **58**: 15863-15868.

[2] Wang S, Wang R and Bian Y *et al*. In-situ encapsulation of pseudocapacitive Li_2TiSiO_5 nanoparticles into fibrous carbon framework for ultrafast and stable lithium storage. *Nano Energy* 2019; **55**: 173–181.

[3] He D, Wang B and Wu T *et al*. TiO_2 Nanocrystal-Framed Li_2TiSiO_5 Platelets for Low-Voltage Lithium Battery Anode. *Adv Funct Mater* 2020; **30**: 2001909.

[4] Liu J, Su D and Liu L et al. Boosting the charge transfer of Li_2TiSiO_5 using nitrogen-doped carbon nanofibers: towards high-rate, long-life lithium-ion batteries. *Nanoscale* 2020; **12**: 19702.

[5] Jin L, Gong R and Zheng J *et al*. Fabrication of dual-modified carbon network enabling improved electronic and ionic conductivities for fast and durable Li_2TiSiO_5 Anodes. *ChemElectroChem* 2019; **6**: 3020–3029.

[6] Jin L, Gong R and Zhang W *et al*. Toward high energy-density and long cycling-lifespan lithium ion capacitors: a 3D carbon modified low-potential Li_2TiSiO_5 anode coupled with a lignin-derived activated carbon cathode. *J Mater Chem A* 2019; **7**: 8234–8244

[7] Liu J, Pang W and Zhou T *et al*. Li_2TiSiO_5: a low potential and large capacity Ti-based anode material for Li-ion batteries. *Energy Environ Sci* 2017; **10**: 1456–1464.

[8] Lim J-M, Kim S and Luu N *et al*. High volumetric energy and power density Li_2TiSiO_5 battery anodes via graphene functionalization. *Matter* 2020; **3**: 522–533.
